# Hydrogel-Based Scaffold Development for Ischemic Stroke In Vitro Platform

**DOI:** 10.1093/geroni/igaf122.4133

**Published:** 2025-12-31

**Authors:** Lidadi Agbomi, Richard Goodwin, Bruce Gao

**Affiliations:** Clemson University, Simpsonville, South Carolina, United States; University of South Carolina, Columbia, South Carolina, United States; Clemson University, Clemson, South Carolina, United States

## Abstract

**Background:**

Age-related neurological disorders, especially cerebrovascular disorders, pose a threat to the ever-increasing geriatric community. Improving tools for clinical studies can aid in the overall outcome of cerebrovascular incidents. In particular, ischemic strokes, a type of cerebrovascular disorder, which is the 2nd leading cause of both death and disability worldwide, can benefit from in vitro platforms that support the development of novel interventions.

**Methods:**

While the microfluidic chip was designed in CAD software, parallel efforts focused on creating the hydrogel scaffold. The first step was choosing the correct material. Material selection was based on desired properties for the scaffold; alginate was selected for its mechanical strength. Considering the tests being done, gelatin was added to help with cell adhesion properties. Following material selection, optimal concentrations were determined. The last test was deciding whether a room-temperature or frozen hydrogel would work best at maintaining the structural integrity of the hydrogel.

**Results:**

A composition of 1% alginate and 5% gelatin, in a ratio of 40/60, was the most suitable hydrogel. Further testing showed that freezing the hydrogel for 15 hours before dropping it into a calcium chloride bath significantly improved structural integrity.

**Conclusion:**

A working hydrogel formulation has been identified, and the next step is to create microchannels within the scaffold for cell culture. Further testing will be done to optimize the materials and techniques to create the channels.

